# Cluster Analysis Revealed Two Hidden Phenotypes of Cluster Headache

**DOI:** 10.3389/fneur.2022.898022

**Published:** 2022-05-20

**Authors:** Pinar Yalinay Dikmen, Cagla Ari, Erdi Sahin, Mustafa Ertas, Fusun Mayda Domac, Elif Ilgaz Aydinlar, Aysenur Sahin, Aynur Ozge, Hilal Ozguner, Omer Karadas, Javid Shafiyev, Doga Vuralli, Cile Aktan, Emel Oguz-Akarsu, Necdet Karli, Mehmet Zarifoglu, Hayrunisa Bolay, Esme Ekizoglu, Elif Kocasoy Orhan, Bahar Tasdelen, Betul Baykan

**Affiliations:** ^1^Department of Neurology, Acibadem University School of Medicine, Istanbul, Turkey; ^2^Department of Neurology, Siirt State Hospital, Siirt, Turkey; ^3^Department of Neurology, Istanbul Faculty of Medicine, Istanbul University, Istanbul, Turkey; ^4^Department of Neurology, Erenkoy Training and Research Hospital for Psychiatric and Neurological Disorders, University of Health Sciences, Istanbul, Turkey; ^5^Department of Neurology, Mersin University Faculty of Medicine, Mersin, Turkey; ^6^Department of Neurology, Gulhane School of Medicine, University of Health Sciences, Ankara, Turkey; ^7^Department of Neurology and Algology, Gazi University Faculty of Medicine, Ankara, Turkey; ^8^Neuroscience and Neurotechnology Center of Excellence (Nörom), Ankara, Turkey; ^9^Gazi University, Neuropsychiatry Center, Ankara, Turkey; ^10^Department of Neurology, Bursa Uludag University, Faculty of Medicine, Bursa, Turkey; ^11^Department of Bioistatistics and Medical Informatics, Mersin University Faculty of Medicine, Mersin, Turkey

**Keywords:** Cluster Headache, cluster analysis, cigarette smoking, tobacco exposure, trigeminal autonomic cephalagia, autonomic features

## Abstract

**Objective:**

To investigate the possible subgroups of patients with Cluster Headache (CH) by using K-means cluster analysis.

**Methods:**

A total of 209 individuals (mean (SD) age: 39.8 (11.3) years), diagnosed with CH by headache experts, participated in this cross-sectional multi-center study. All patients completed a semi-structured survey either face to face, preferably, or through phone interviews with a physician. The survey was composed of questions that addressed sociodemographic characteristics as well as detailed clinical features and treatment experiences.

**Results:**

Cluster analysis revealed two subgroups. Cluster one patients (*n* = 81) had younger age at diagnosis (31.04 (9.68) vs. 35.05 (11.02) years; *p* = 0.009), a higher number of autonomic symptoms (3.28 (1.16) vs. 1.99(0.95); *p* < 0.001), and showed a better response to triptans (50.00% vs. 28.00; *p* < 0.001) during attacks, compared with the cluster two subgroup (*n* = 122). Cluster two patients had higher rates of current smoking (76.0 vs. 33.0%; p=0.002), higher rates of smoking at diagnosis (78.0 vs. 32.0%; p=0.006), higher rates of parental smoking/tobacco exposure during childhood (72.0 vs. 33.0%; *p* = 0.010), longer duration of attacks with (44.21 (34.44) min. vs. 34.51 (24.97) min; p=0.005) and without (97.50 (63.58) min. vs. (83.95 (49.07) min; *p* = 0.035) treatment and higher rates of emergency department visits in the last year (81.0 vs. 26.0%; *p*< 0.001).

**Conclusions:**

Cluster one and cluster two patients had different phenotypic features, possibly indicating different underlying genetic mechanisms. The cluster 1 phenotype may suggest a genetic or biology-based etiology, whereas the cluster two phenotype may be related to epigenetic mechanisms. Toxic exposure to cigarettes, either personally or secondarily, seems to be an important factor in the cluster two subgroup, inducing drug resistance and longer attacks. We need more studies to elaborate the causal relationship and the missing links of neurobiological pathways of cigarette smoking regarding the identified distinct phenotypic classes of patients with CH.

## Introduction

Cluster Headache (CH) is the most common form of trigeminal autonomic cephalalgia and is characterized by short-lasting attacks of excruciating unilateral headache associated with ipsilateral autonomic features and/or restlessness or agitation (1). The diagnosis of CH is firmly based on clinical history because of the lack of a diagnostic marker. The prevalence of CH is estimated at 0.5–3/1000, with a male predominance (2). Even though CH is rare compared with migraine, more than 500,000 individuals are probably experiencing this “suicidal” primary headache syndrome in the United States of America (USA) alone (3).

The complex neurobiologic mechanism underlying CH remains incompletely understood. Hypothalamic activation along with secondary activation of the trigeminal-autonomic reflex is the leading hypothesis in the pathophysiology of CH (4–7). Several important questions have yet to be solved, such as those regarding seasonal differences, the reasons for transformation from episodic form to a chronic form, sex differences, regional differences between Asian and Western countries, responsible genes in the development of CH, and a visible link with cigarette smoking/tobacco exposure and CH (8–24).

Higher rates of cigarette smoking of patients with CH as well as in their parents have been reported (25, 26). Although there is no strong evidence between quitting smoking and the improvement of CH, the association between cigarette smoking and CH is very convincing (27, 28). Personal cigarette smoking history and secondhand tobacco exposure during childhood are both important factors leading to CH onset and its clinical presentations, based on the USA Cluster Headache Survey (8, 25, 26). Individuals with CH who had secondhand exposure to cigarette smoking during their childhood, regardless of whether they smoked later in their lives, had 2.5 times increased risk for developing CH in their younger ages compared with non-exposed patients with CH (25, 26).

In this study, we aimed to investigate CH from a different perspective. Hierarchical unsupervised k-cluster analysis was used to group patients who were diagnosed as having CH by headache experts. Cluster analysis aims to identify subgroups of patients with CH to suggest some clues for underlying mechanisms as well as for management issues. As far as we know, this statistical analysis method has not been used previously to classify patients with CH. Our purpose was to reveal which factors came together to create possible subgroups of patients with CH using the cluster analysis. Thus, the aim of the study was to explore any hidden groups in a large population with CH.

## Materials and Methods

### Study Population

**Figure 1:**
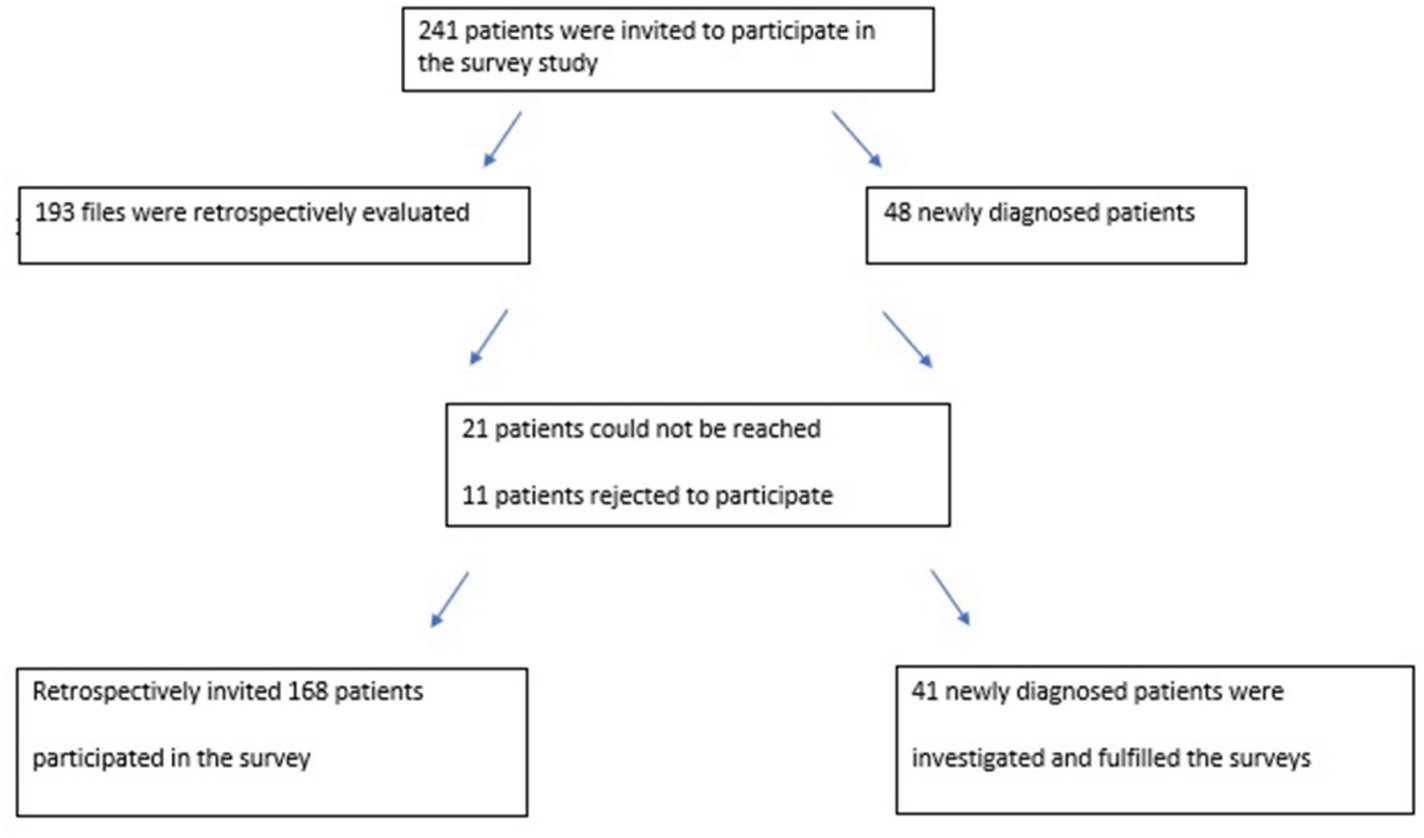
A flow chart of the study design.

The study was a cross-sectional investigation (performed between January and June 2020) based on data from eight tertiary headache centers in Turkey. Participants were recruited from the headache centers in two ways. First, patients diagnosed as having CH were screened retrospectively in the records of the headache centers. Then, they were invited by phone to participate in the study. A total of 168 registered patients with CH volunteered to participate in this study. Secondly, patients who were newly diagnosed as having CH were also enrolled from the outpatients or emergency departments (ED) of these centers during the recruitment period, and 41 participants with episodic CH were enrolled in the study in this way. Only 11 individuals rejected to participate in the study. We could not reach 21 registered patients with CH either by phone or email during the study (Figure 1). All included patients were re-evaluated by experienced headache specialists and their diagnoses of CH were checked according to the International Classification of Headache Disorders-3 criteria (1).

Written informed consent was obtained from each subject following a detailed explanation of the objectives and protocol of the study that was conducted in accordance with the ethical principles stated in the Declaration of Helsinki. Institutional review board approval was granted by Acibadem University School of Medicine.

### Inclusion and Exclusion Criteria

Inclusion criteria for the study were accepting to participate in the study and being diagnosed with episodic CH (ECH) or chronic CH (CCH) by a headache expert. Exclusion criteria were a diagnosis of secondary CH, unwillingness to participate, illiteracy, and unstable medical and psychiatric conditions.

### Assessments

All patients completed a semi-structured survey, either face to face, preferably, or by phone interview with a physician, due to the restrictions related to the pandemic. The survey was composed of questions that addressed sociodemographic characteristics as well as detailed clinical features, delay of diagnosis, triggers for attacks, and treatment experiences (Appendix 1). The majority of the questions were adopted from the USA Cluster Headache Survey (8). The participating eight headache centers were in five out of seven different geographic regions in Turkey (Marmara, Aegean, Mid-Anatolian, Mediterranean, and South-East Anatolian regions).

### Statistical Analysis

This is the primary analysis of these data. No *a priori* statistical power calculation was conducted. The sample size was based on the available data. There were missing values on some variables; therefore, the percentages were calculated from valid cases. The normality of data distribution was evaluated using the Shapiro-Wilk test. Hypothesis testing was two-tailed. Data are expressed as mean (SD) and median (minimum-maximum), count, and percentages (*n*, %). *P* < 0.05 was considered statistically significant. The Chi-square (χ^2^) test, Yates continuity correction, and Fisher's exact test were used for the comparison of categorical data. Numerical data were analyzed using the Mann-Whitney U test for non-normally distributed two-group comparisons, whereas the Kruskal-Wallis test was used for the comparison of more than two variables that were non-normally distributed. Post hoc pairwise comparisons were performed using a Bonferroni-corrected Mann-Whitney U-test. The patients who were current smokers and exposed to tobacco during their childhood were included to create “smokers” group (*n* = 154).

K-means clustering is the most known unsupervised machine learning algorithm that uses categorical and continuous variables together. K-means clustering is described simply as a method of vector quantization, originally from signal processing, that aims to partition n observations *k* clusters, in which each observation belongs to the cluster with the nearest mean (cluster centers or cluster centroid), serving as a prototype of the cluster analyses. K-means cluster analysis for this study was accomplished by one of our authors (BT) and the variables were selected by headache experts regarding both available demographic, clinical and treatment experiences of the patients diagnosed with CH. These variables were also in accordance with related publications on CH. Clinical and demographic features are form of CH (episodic vs. chronic), gender, age, the duration of CH, the duration of diagnostic delay, previous diagnosis with other conditions (previously diagnosed as migraine), a history with head trauma, being an active smoker, being a smoker at the time of diagnosis of CH, tobacco exposure during the childhood, and alcohol consumption. Clinical attack features are the frequency of the daily attacks, the length of attack duration, number of autonomic features during an attack.Treatment experiences are responsiveness to triptan, responsiveness to oxygen treatment, responsiveness to attack treatment, and an admission to the ED in the previous past year.

We explained the k-means algorithm details, and steps as below. Input variables (categorical and continuous) were determined by the headache expert team. Initial cluster centers were specified maximizing the initial cluster distances. The number of clusters was determined automatically from the data using 10-fold cross-validation. All objects were assigned to the nearest centroid using standardized Euclidean distance. The new centroids for both continuous and categorical variables were recalculated. If all the observations were assigned to the same cluster as the earlier iteration, the iteration was stopped.

In this study, minimum cluster size was 81 and statistical power was enough according to earlier simulation studies (29). The authors explained that when the effect size between two clusters with relatively small samples (*n* ≥ 20) was large (Δ = 4), sufficient (>90%) statistical power was achieved. They also noted that, for clustering algorithms, if there was a clear separation between clusters, power was satisfactory (29).

The validity of the results of cluster analysis was assessed by comparing the groups in which common features were clustered. To control familywise error rates in these comparisons regarding the main hypothesis of the study, Bonferroni adjustment was used. Moreover, k-means cluster analyses have the advantage of not requiring any imputation method for missing values compared with standard statistical techniques. It is simply based on the density estimation of variables that are observed for the case concerned. Statistical analysis was performed using the STATISTICA 13.0 software package (TIBCO Software Inc.).

## Results

A total of 209 individuals with a mean age of 39.8 (11.3) (range: 18–71) years completed the survey. There were 176 males (84.2%) and 33 females (15.8%), and 188 of them had ECH (88.5%) whereas the remaining 21 had CCH (11.5%). The mean age at CH onset was 28.6 (10.2) years. The mean age at the correct diagnosis was 33.5 (11.1) years. In our cohort, the mean diagnostic delay was 4.9 (6.3) years.

**Figure 2:**
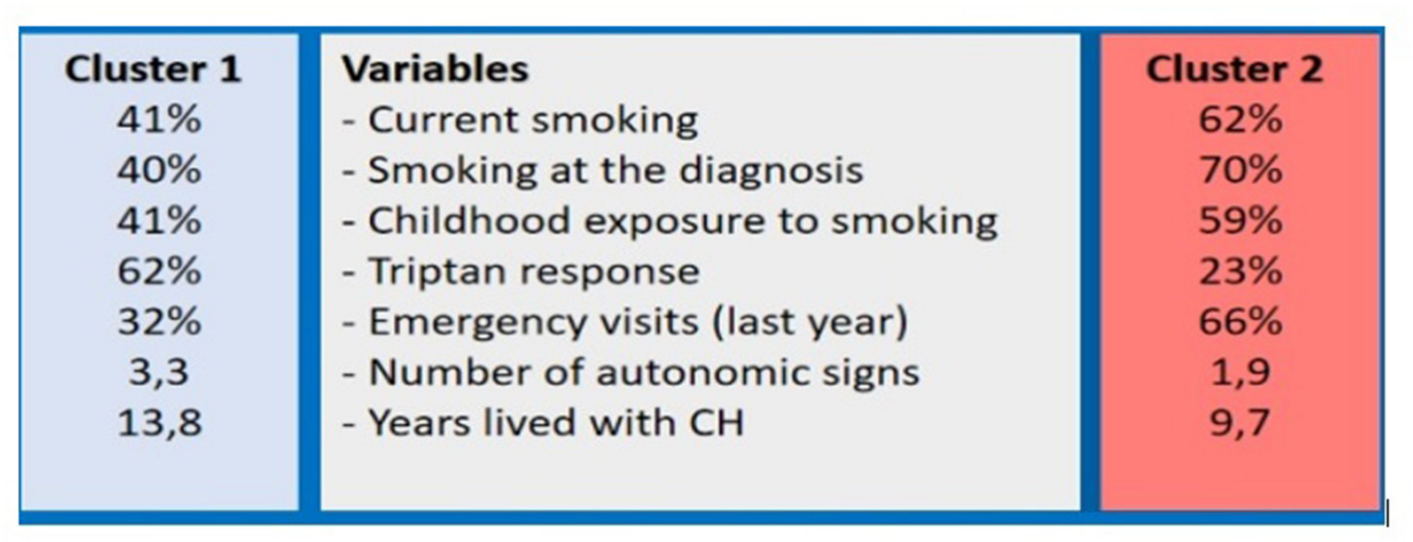
The cluster 1 and 2 patients with Cluster Headache.

After the input of the indicated variables above, two distinct subgroups of patients with CH were identified (Figure 2). Table 1 shows the demographic characteristics of these patients. Cluster one patients were younger compared with the cluster two group at the diagnosis (31.04 (9.68) vs. 35.05 (11.32) years; *p* = 0.009). Years lived with CH were longer in cluster 1 group (13.78 (9.36) vs. 9.67 (8.17); *p* = 0.001). Cluster two patients displayed higher rates for being current smokers in comparison with cluster one (62.30 vs. 40.74%; *p* = 0.002) and they also had a significantly longer history of smoking at the time of correct diagnosis (63.93 vs. 39.51%; *p* = 0.006). Cluster two patients had parental secondary smoke exposure in childhood compared to cluster one patients (59.02 vs. 40.74%; *p* = 0.010).

**Table 1:** Demographic characteristics of the patients in cluster analysis.

**Variables**		**Cluster 1**	**Cluster 2**	***p***
		**(*n* = 81)**	**(*n* = 122)**	
Age (years)	Mean (SD)	39.68 (10.72)	39.85 (11.39)	0.913
CH diagnosis history (years)*	Mean (SD)	13.78 (9.36)	9.67 (8.17)	**0.001**
Age at the diagnosis	Mean (SD)	31.04 (9.68)	35.05 (11.32)	**0.009**
Diagnostic delay (years)*	Mean (SD)	5.14 (5.89)	4.87 (6.64)	0.763
Smoking (years)	Mean (SD)	13.93 (10.65)	14.37 (10.08)	0.812
Type of CH	Episodic *n* (%)	74 (91.36)	109 (89.34)	0.634
	Chronic *n* (%)	7 (8.64)	13 (10.66)	
Sex	Female *n* (%)	16 (19.75)	16 (13.11)	0.207
	Male *n* (%)	65 (80.25)	106 (86.89)	
Previous diagnosis with other conditions	*n* (%)	47 (58.02)	70 (57.38)	0.927
Previous diagnosis with migraine	*n* (%)	29 (35.80)	36 (29.51)	0.347
Previous history with head trauma	*n* (%)	9 (11.11)	11 (9.02)	0.625
Alcohol consumption	*n* (%)	39 (48.15)	47 (38.52)	0.175
Current smoking	*n* (%)	33 (40.74)	76 (62.30)	**0.002**
Smoking at the diagnosis	*n* (%)	32 (39.51)	78 (69.93)	**0.006**
Parental smoking/tobacco exposure during childhood	*n* (%)	33 (40.74)	72 (59.02)	**0.010**

*N, number of subjects; SD, Standard deviation; CH, Cluster Headache; Bold indicates p < 0.05*;

* *Mann-Whitney U-test was used to compare differences, not normally distributed*.

Table 2 demonstrates autonomic features ipsilateral to the headache, and associated symptoms during CH attacks. Cluster 1 patients had more conjunctival injections (66.67 vs. 46.72%; *p* = 0.004), lacrimation (90.12 vs. 72.13%; *p* = 0.001), miosis and/or ptosis (71.60 vs. 34.43%; *p* < 0.001), nasal congestion (74.07 vs. 41.80%; *p* < 0.001), rhinorrhea (69.14 vs. 27.87%; *p* < 0.001), forehead and facial sweating (34.57 vs. 18.85%; *p* = 0.012), and agitation (74.07 vs. 40.16%; *p* < 0.001). Migrainous features such as nausea and/or vomiting (40.74 vs. 27.05%; *p* = 0.042) and phonophobia (22.22 vs. 7.38%; *p* = 0.002) during attacks were mostly reported by the cluster one group.

**Table 2:** Associated symptoms or signs, ipsilateral to the headache, which were reported by the patients in cluster analysis.

**Variables**		**Cluster 1**	**Cluster 2**	***p***
		**(*n* = 81)**	**(*n* = 122)**	
Conjunctival injection	*n* (%)	54 (66.67)	57 (46.72)	**0.004**
Lacrimation	*n* (%)	73 (90.12)	88 (72.13)	**0.001**
Miosis and/or ptosis	*n* (%)	58 (71.60)	42 (34.43)	**<0.001**
Nasal congestion	*n* (%)	60 (74.07)	51 (41.80)	**<0.001**
Rhinorrhea	*n* (%)	56 (69.14)	34 (27.87)	**<0.001**
Forehead and facial sweating	*n* (%)	28 (34.57)	23 (18.85)	**0.012**
Agitation	*n* (%)	60 (74.07)	49 (40.16)	**<0.001**
Nausea and/or vomiting during attacks	*n* (%)	33 (40.74)	33 (27.s05)	**0.042**
Phonophobia during attacks	*n* (%)	18 (22.22)	9 (7.38)	**0.002**
Vertigo during attacks	*n* (%)	6 (7.41)	6 (4.92)	0.465

*n, number of subjects; Bold indicates p < 0.05*.

Table 3 shows differences in clinical features and treatment experiences of the patients in the cluster analysis. Duration with (44.21 (34.44) min vs. 34.51 (24.97) min; *p* = 0.005) or without (97.50 (63.58) min vs. 83.95 (49.97) min; *p* = 0.035) treatment were longer in the cluster two group. The number of autonomic symptoms during attacks was statistically higher in cluster one patients (3.28 (1.16) vs. 1.99 (0.95); *p* < 0.001). Treatment experiences were more favorable in cluster one patients. They were more patients with response to triptans (50.0 vs. 28.0%; *p* < 0.001) and attack treatment (59.26 vs. 30.33%; p>0.001). Higher rates of ED visits in the last year were reported by cluster 2 patients (26.0 vs. 81.0%; *p* < 0.001).

**Table 3:** Clinical features and treatment experiences of the patients in cluster analysis.

**Variables**		**Cluster 1**	**Cluster 2**	***p***
Duration of attack with treatment*	Mean (SD)	34.51 (24.97)	44.21 (34.44)	**0.005**
Duration of attack without treatment*	Mean (SD)	83.95 (49.07)	97.50 (63.58)	**0.035**
Number of autonomic symptoms*	Mean (SD)	3.28 (1.16)	1.99 (0.95)	**<0.001**
Number of attacks per day	1–2	29 (35.80)	54 (44.26)	0.390
	3–5	37 (45.68)	52 (42.62)	
	>5	15 (18.52)	16 (13.11)	
Oxygen was effective for attacks	*n* (%)	27 (33.33)	54 (44.26)	0.117
**Triptan was effective for attacks**	*n* (%)	50 (61.73)	28 (22.95)	**<0.001**
**Mostly responsive to attack treatment**	*n* (%)	48 (59.26)	37 (30.33)	**<0.001**
**ED visit at the last year**	*n* (%)	26 (32.10)	81 (66.39)	**<0.001**

*n, number of subjects; SD, Standard deviation; bold indicates p < 0.05; ED, emergency department*;

* *Mann-Whitney U-test was used to compare differences, not normally distributed*.

Table 4 illustrates the demographic, clinical features and treatment experiences of the smokers (*n* = 154), and the non-smokers (*n* = 55), comparatively. In our cohort, 73.4% of the participants was active smokers (*n* = 114) and 74.0% (*n* = 113) was smokers at the time of diagnosis. Regarding parenteral tobacco exposure, 71.4% of the participants (*n* = 110) reported that one of their parents was active smoker at home during their childhood. A mean duration of smoking was reported as 15.44 (9.63) years.

**Table 4:** Comparison of two groups who never smoked vs. those who smoked/exposed.

**Variables**	**Smokers (*n* = 154)**	**Nonsmokers (*n* = 55)**	***p***
Age (Mean, SD)	40.64 (11.43)	37.55 (10.64)	0.073
Female/Male (*n*,%)	19 (12.3%) 135 (87.7%)	14 (25.5%) 41 (74.5%)	**0.030**
Episodic Cluster Headache (*n*,%)	135 (87.7%)	50 (90.9%)	0.627
Chronic Cluster Headache (*n*,%)	19 (12.3%)	5 (9.1%)	
Age at onset (years) (Mean, SD)	28.66 (10.74)	28.27 (8.84)	0.795
Age at correct diagnosis (years) (Mean, SD)	33.84 (11.64)	32.52 (9.71)	0.411
Family history with CH (*n*, %)	17 (11.0%)	2.0 (3.6%)	0.169
Family history with heart diseases (*n*,%)	65 (42.2%)	5 (9.1%)	**<0.001**
Head trauma before the diagnosis (*n*,%)	21 (13.6%)	0.0 (0%)	**0.001**
NAS (Mean, SD)	8.78 (1.3)	8.58 (1.23)	0.316
Nasal congestion (*n*,%)	92 (59.7%)	23 (41.8%)	**0.027**
Pain location (behind eyes) (*n*,%)	139 (90.3%)	50 (90.9%)	0.564
Pain location (temple) (*n*,%)	107 (69.5%)	28 (50.9%)	**0.021**
Improvement with both O2 and triptan (*n*,%)	62 (52.1%)	14 (32.6%)	**0.033**

*n, number of subjects; SD, Standard deviation; CH, Cluster Headache; NAS, Numeric Analog Scale; O, Oxygen, bold means p < 0.05;*

* *Unpaired t-test*.

## Discussion

Our cluster analysis resulted in the identification of two remarkable subgroups of CH, varying considerably concerning the age at diagnosis, cigarette smoking, autonomic signs and symptoms, associated features during attacks, and treatment experiences. These clusters can be conveniently characterized as follows: the first group includes uncomplicated patients with CH who had longer disease duration, higher rates of autonomic signs and symptoms, associated migraine-like features, and more favorable treatment experiences (cluster 1), and the second cluster comprised of complicated patients who had more prominent exposure to cigarette smoking, fewer autonomic signs and symptoms, longer duration of attacks with or without medication, and higher rates of ED visits in the last year (Cluster 2).

Patients in cluster one vs. cluster two had different phenotypic features, possibly indicating differing underlying mechanisms. Patients in the cluster one subgroup reported higher rates of drug responsiveness for their acute attacks despite having a longer disease duration and a higher number of autonomic signs and symptoms. We thought that cluster one might be classified as patients who had a characteristic primary headache disorder without or less exposure to any negative environmental factors (head trauma, consequences of a negative lifestyle, obesity, coffee consumption) and toxins (30–32). Therefore, cluster one patients may suggest a genetic or biology-based etiology. In our study, patients in cluster two had a prominent personal history with cigarette smoking and parental secondary smoke exposure as children. The duration of their attacks was longer with or without medication compared with cluster one patients and they reported higher use of ED during the last year. Thus, the cluster two phenotype seems to have a different form of the disease, possibly related to remote environmental mechanisms, compared with the cluster one phenotype. However, the comparison of the smokers and the non-smokers in this study did not show any difference in relation to the age, age at onset, diagnostic delay, NAS, clinical features of attacks, treatment responses to triptans and oxygen. As expected, male predominance was shown in the smokers (87.7 vs. 12.3%). They were exposed to tobacco in their childhood (71.4 vs. 0.0%; *p* < 0.001) compared to the non-smokers. Rates of smoking during the diagnosis (74.0%), and active smoking during the recruitment of study (73.4%) showed that the smokers did not quit smoking, while their physicians probably suggested stopping smoking based on scientific data so far.

In our study, cluster analysis showed that sex and subtypes of CH (episodic vs. chronic) did not play a role in the determination of subgroups. Other possible demographic determinants such as diagnostic delay, previous misdiagnosis, having a minor head trauma, or alcohol intake also did not contribute to the identification of these subgroups. However, cigarette smoking and tobacco exposure during childhood played a role in belonging to the cluster two phenotype. Moreover, the comparison of smokers and non-smokers showed that the length of education was statistically shorter in the smokers (13.63 vs. 15.15 years; *p* = 0.01). The smokers had more likely to have a history of head trauma and heart diseases in their family, and a previously wrong diagnosis with sinusitis. Environmental factors may play a role to reveal and prognosis estimation of CH.

It is well-known that CH is strongly linked to cigarette smoking (26, 28, 30, 33–39). However, a satisfactory explanation of this connection has not yet been established. Recently, Rozen proposed a detailed theoretical background to establish a relationship between cigarette smoking and the pathogenesis of CH (26). The hypothesis was mainly dependent on findings from the USA Cluster Headache Survey (8, 25, 26). Results from the survey showed that 12% of the surveyed population (n=133) had no personal smoking/tobacco use history and no parental smoking exposure during their childhood. In these nonexposed participants, the male to female (M/F) ratio was 1.9:1 and 80% of them had ECH. These patients were more likely to develop CH at ages 40 years and younger. On the other hand, the M/F ratio was 2.7:1 in an exposed survey population (*n* = 1001, 88%) who had a personal smoking history (83%) and parental smoking exposure (17%). The double exposure rate (personal smoking history and secondary exposure as a child) was reported as 85%. Remarkably, these exposed participants had more severe CH based on attack frequency, cycle duration, and headache-related disability. In parallel with our results, Rozen previously suggested that the underlying pathology might be different between non-exposed and exposed patients with CH (26). Indeed, the findings from this former large survey are compatible with our clinical results, which emerged from a different population with CH by using cluster analysis, for the first time. However, our dichotomic analysis with the smokers and non-smokers did not show any difference in the beginning age of the disease or the clinical features. As a remarkable finding, combined usage of triptan and oxygen was more likely to be reported by the smokers compared to the non-smokers in our study (52.1 vs. 32.6%; *p* = 0.03). A classical location of the pain is behind the eye in patients with CH. Our study showed that the location of pain in the temple was also statistically higher in smokers. The smokers might experience wider distribution of pain than the non-smokers. These latter findings might be interpreted as increased severity of the disease.

Remarkably, the non-smoking-exposed group in the USA survey in whom disease developed at younger ages seems to be compatible with our cluster one phenotype, which also had a longer disease duration. The cluster one phenotype had more benign disease progress, they were more responsive to triptans and other acute treatment. In the US survey, nonexposed participants had a higher frequency of migraine family history. In agreement, our cluster one phenotype had some migrainous features such as nausea and phonophobia. It is tempting to speculate that the cluster one phenotype may mainly bear a genetic-based etiology (25, 26). The cluster two phenotype (personal and/or parental tobacco exposed) may also have susceptibility factors (probably genetic), then tobacco exposure emerges to be a trigger factor that leads to CH onset. In our study, both current smoking, smoking at diagnosis, and parental smoke exposure during childhood clustered in the cluster two phenotype (*n* = 123). These cluster two patients had longer attack durations and higher rates of ED visits in the past year like exposed patients, who had a severe form of CH, in line with the USA survey.

Recent findings from a Korean CH study were also compatible with these findings. The authors aimed to determine clinical differences in features between patients with a smoking history and those who were never-smokers (37). In this prospective multicenter study, 60.8% of patients with CH (*n* = 152) were ever-smokers and 39.2% (*n* = 98) were never-smokers. Similar to our results, the age of CH onset was notably lower in the never-smoker group compared with the ever-smoker group (27.1 (12.9) years vs. 30.6 (10.9) years; *p* = 0.024). Moreover, triptan responsiveness was higher in the never-smoker group (100 vs. 85.1%; *p* = 0.001). They also suggested that smoking acted as a secondary environmental contributor to CH generation, supporting our results.

The rates of being a previous or current smoker were high in patients with CH, as 73–90% (8, 26–28, 30, 33–39). In this study, the percentage of active smokers in all patients with CH (54.1%) was higher than the average rate of overall active smokers (29.3%) in Turkey (2018) (40). Our male patients with CH had statistically higher rates of current (59.7% vs. 24.2; p<0.001) smoking and smoking at diagnosis (60.8 vs. 21.2; *p* < 0.001) compared with females, in line with the previous studies (8). However secondhand cigarette smoke exposure during childhood was not statistically different between our male and female patients (53.5 vs. 48.5; *p* = 0.705). Interestingly, sex did not appear in the identification of cluster subgroups in our study. This finding may indicate that smoking history is a more reliable marker than sex itself. In this study, our results showed that the parents of the patients with CH who are smokers were more likely to be smokers. From this point of view, children who are exposed to toxic chemicals from cigarette smoking may be more susceptible to the development of CH due to an alteration of hypothalamus-based neurotransmitter function.

Taken together, exposure to the toxic effect of cigarette smoking might play a role in the transformation of clinical features in patients with CH who have a genetic susceptibility. Cigarette smoking/tobacco exposure during childhood may transform the disease into a severe form. Studies showed that current smokers had higher numbers of attacks with longer bouts than patients with CH who reported never having smoked (8, 27).

In terms of autonomic symptoms, cluster one phenotype reported more autonomic signs and symptoms than cluster two phenotype. The tobacco-exposed participants were more likely to experience cranial autonomic symptoms in the US Cluster Headache Survey (8). Their explanation of this finding might be related to tobacco exposure possibly leading to excessive sphenopalatine/trigeminal autonomic pathway activation in patients with CH (26). Therefore, our findings were not compatible with the USA survey in this regard. In a recent study from Korea, the authors found no relationship between symptoms' severity and cigarette smoking, most of the clinical findings did not differ significantly between ever-smokers and never-smokers (38). These differences might reflect some unknown genetic features between different races. We may speculate that cluster one phenotype is related to a pertinent genetic liability to CH with a stronger association of autonomic symptoms. On the other hand, cluster type two phenotype reflected another severe and drug-resistant variant which was induced by toxic triggers in a different genetic background but was less prone to autonomic symptom induction. As expected, the smokers had higher rates of nasal congestion compared to the non-smokers (59.7. vs. 41.8%; *p* = 0.027).

Several limitations are present in this study. First, our cohort was hospital-based. For that reason, our findings and conclusions may not be generalized to community-based patients. Secondly, we collected data retrospectively from patients' files and interviews. Hence, recall bias may obscure our results (41, 42). In this study, we investigated current smoking, smoking at diagnosis, and parental smoke exposure during childhood. We did not search for detailed information about smoking habits such as the duration and the amount of cigarette smoking. We have statistically different rates of patients who had a personal or parental smoking history in both cluster groups. It is something to keep in mind that the smokers had different types of smoking exposures like current active ones, those who have quit many years ago, those who smoked shorter terms or longer terms, or those exposed only in the family, etc. Therefore, we grouped only those who never smoked/exposed vs. the remaining patients. We believe that cluster analysis was valuable to identify hidden groups. Thirdly, the coexisting migraine diagnoses in our cohort may create problems. This comorbid condition might blur some of our results. Nevertheless, the study has some obvious strengths. This is the first large-sized multicenter study about CH from Turkey and our findings were gathered through face-to-face or detailed phone interviews due to the pandemic by experienced headache specialists. To the best of our knowledge, this is the first study to use cluster analysis for identifying distinct hidden CH phenotypes in patients with CH.

Cluster one and two patients with CH seem to appear concerning biology-based etiology and environmental influences (the consequence of smoke exposure), respectively. Although the presence of familial clustering of CH suggests a genetic predisposition, the genetic background is far from understood. Two recent genome-wide association studies reported seven loci (the same 4 loci in both studies) associated with CH. The existing evidence regarding the genetics of CH gave rise to a hypothesis of polygenic predisposition. In other words, environmental risk and triggering factors such as smoking in CH could contribute to the disease mechanism as well. Cluster analysis can be used to identify subgroups of patients with unique characteristics. This is accomplished by assessing similarities between patients. A better understanding of the sources of heterogeneity may lead to more effective treatment strategies according to patient profiles. We think that our approach with cluster analysis will be of help in unraveling underlying genetic mechanisms of CH, which seems to bear some heterogeneity within its clinical phenotype (43, 44).

In conclusion, future prospective studies are needed to elucidate the causal relationship and the missing links of neurobiological pathways of cigarette smoking exposure regarding the identified distinct phenotypic classes of patients with CH.

## Data Availability Statement

The original contributions presented in the study are included in the article/Supplementary Material, further inquiries can be directed to the corresponding author/s.

## Ethics Statement

The studies involving human participants were reviewed and approved by Acibadem University School of Medicine. The patients/participants provided their written informed consent to participate in this study.

## Author Contributions

PY, BB, ME, and BT had full access to all the data in the study and take responsibility for the integrity of the data and the accuracy of the data analysis. PY and BB: study concept and design and drafting of the manuscript. PY, CA, ES, ME, FM, EI, AS, AO, HO, OK, JS, DV, CA, EO-A, NK, MZ, HB, EE, EK, and BB: acquisition of data. PY, BB, AO, ME, and BT: analysis and interpretation of data. PY, BB, AO, and HB: revising it for intellectual content. All authors contributed to the article and approved the submitted version.

## Conflict of Interest

The authors declare that the research was conducted in the absence of any commercial or financial relationships that could be construed as a potential conflict of interest.

## Publisher's Note

All claims expressed in this article are solely those of the authors and do not necessarily represent those of their affiliated organizations, or those of the publisher, the editors and the reviewers. Any product that may be evaluated in this article, or claim that may be made by its manufacturer, is not guaranteed or endorsed by the publisher.
