# Supplementary material for: Cluster Analysis Revealed Two Hidden Phenotypes of Cluster Headache
Source: Front Neurol. 2022 May 20;13:898022. doi: 10.3389/fneur.2022.898022 (PMC9163308; doi:10.3389/fneur.2022.898022)
Supplement: Supplementary file 1 [file Data_Sheet_1.doc]

**Cluster Headache Questionnaire**

**Type of Cluster Headache**: Episodic/ Chronic

**Demographic Data**

- Full Name

- Phone number

- Gender

- Date of Birth

- Age

- Educational attainment (… years)

- Occupation

**Cigarette Smoking**

a) Are you currently an active or passive smoker?

b) Were you a smoker when you were diagnosed with Cluster Headache (CH)?

c) Did your parents smoke when you were a child?

d) How many years total have you smoked cigarettes? (pack/years)

**Alcohol consumption uses**

- How often do you have a beverage containing alcohol per week?

- What kind of alcoholic beverages do you usually consume?

- Which amount alcohol do you usually consume per week?

**Please list** any medical conditions that you have)

- Hypertension, diabetes, cardiac disease, hypercholesterolemia, stroke, migraine, any other headache disorder (trigeminal neuralgia, etc.), restless leg syndrome, depression, anxiety disorder, ulcer/gastritis, sleep apnea, other sleep disorders, other diseases

**Please list** any regular medications you require (include dosage)

**Family history**

- Has any member of your family been diagnosed with cluster headache?

- If the answer is yes, who was diagnosed?

- Has any member of your family been diagnosed with any cardiac disease?

-Does your mother and/or father have headaches history (migraine or other types)?

- Did you have a head injury before your cluster headache started?

**Clinical Characteristics of Cluster Headache**

- How long have you been suffering from CH?

- What age did your CH start?

- What age were you diagnosed with CH?

- Which practitioner diagnosed you with CH?

- Which of the following specialist did you diagnose your CH?

Neurologist, general practitioner, internist, dentist, otolaryngologist, neurosurgeon, other

- Please circle the followings if you were misdiagnosed any of them before you were diagnosed with CH

Sinusitis, dental diseases, migraine, allergic diseases, other

- When was your last cluster headache bout?

- Does headaches on the same side of your head in every cluster headache period? (Diagnosis confirmation)

- Does your headache always choose to right or left-side of your head on during your bout? (Yes/ No)

- If your answer is yes, which side? (right/left)

- Does your headache change side during your attack?

- Does your headache change side during different attacks in the same bout?

- Does your headache change side during different bouts?

- Please circle if you have any following symptoms during CH attack?

- Conjunctival injection and/or tearing lacrimation

- Nasal congestion

- Rhinorrhoea

- Eyelid oedema

- Forehead

- Facial sweating

- Myosis

- Ptosis

- Agitation

- On scale of 0 to 10, how do you scale your headache intensity during CH attack? (0: no pain, 10: worst headache of your life) (Numeric Analogue Scale)

- If you also have any following symptoms during CH attacks

Nausea, vomiting, dizziness, odor sensitivity, vertigo

- How long does your headache continue? (If you take your medicine; …… minutes, if you don’t take any medicine; .…… minutes)

- Which location of your head do you feel the pain most? (Can be multiple areas of head)

Behind the eyes, around eyes, forehead, temples, back of the neck, ear, shoulder, upper dental area, jaw

- Which of the followings do you trigger for your CH attack?

a) Alcohol b) Sleep disturbances c) Stress d) Seasonal changes d) Weather conditions e) Odor f) Hot shower g) Medications h) Menstruation ı) Other (other drugs, infection, food etc.)

- How many headache attacks occur per day during your CH bout? (Please indicate number)

- Do your attacks occur generally at the same hours of day? Yes/No

- Do these attacks usually occur during the daytime or the nighttime of day?

- Do these attacks occur at a special time of the day? (Please give details)

- Do you find yourself damaging behavior during the attack?

- If yes, what are you doing?

**About bouts**

- How often do you have cluster headache bout per year?

- How many days/weeks does your CH bout last? (weeks)

(With prophylactic medication /without prophylactic medication)

- In which months do you usually have your cluster bouts?

- Do your cluster bouts trigger by seasonal changes? (Yes/ No)

- If your answer is yes, which special time of the year your headache usually occurs?

a) from winter to spring

b) from spring to summer

c) from summer to autumn

d) from autumn to winter

**Radiological Imaging**

How many cranial imaging have been performed so far because of the headache?

…Cranial Tomography, ...Magnetic Resonance Imaging (MRI)

Results of your head MRI are normal or insignificant

**Treatment**

- Which of the following do you use for CH attacks?

- Which of the following medications did you usually use for your headache attacks?

a) Paracetamol

b) Non-steroidal anti-inflammatory drugs

c) Triptans

d) Oxygen

e) None

f) Other (Metoclopramide, lidocaine, etc.)

- Which methods do you think is most effective for stopping your CH attack? (medicine, massage, oxygen, sleep, etc.) (Multiple choices might be possible. Please give names of medicine which you mention)

- What do you think about efficacy of drugs which you experienced about abortive treatment of CH? They were usually effective to control your attacks (Yes/No)

- Which abortive medication do you think is most effective for controlling your CH attack?

- Which of the following did you use/try for your CH attack?

a) Subcutaneous Sumatriptan b) Oral Sumatripran c) Eletriptan d) Oral Zolmitriptan e) Rizatriptan

- Have you ever taken any prophylactic treatment for CH?

- Which of the following prophylactic medication did you use for CH treatment? (Please indicate dosages if possible)

a) Verapamil b) Lithium c) Cortisone d) Valproic acid e) Flunarizine f) Topiramate g) Melatonin h) Botulinum toxin i) Other

- Have you been using any prophylactic medicine when you are answering this questionnaire?

- If any, which drug?

- Do you use non-steroidal anti-inflammatory drugs or triptan for every day?

- If your answer is yes, how many pills do you take for your headache per day?

- For how long do you take these attack medications?

- Which of the following have you tried as an alternative treatment method for CH?

1. Acupuncture b) Herbal medicine c) Ganglion blockage d) Other

- How many times did you have to go to the Emergency Department in the last one year due to your headache attack?

**Oxygen**

- Have you ever used Oxygen for cluster headache attack treatment?

- If your answer is yes, which the specialist that suggested to use Oxygen for your cluster headache attack treatment

Neurologist/Emergency physician/Family practitioner/ Other

- Do you think Oxygen treatment is effective for treating your headache attacks? Yes/No

- How long does it take for the attacks to end with Oxygen therapy?

- Do you usually need to use oral medication in addition to Oxygen therapy?

- How many times did you use Oxygen treatment?

- Where did you take Oxygen treatment?

a) Hospital b) House c) Both

- Do you have an Oxygen tube cylinder at your home?

- Did any physician advice to buy an Oxygen cylinder for home?

- Do you usually use Oxygen with a facial mask or a nasal cannula during Oxygen therapy?

- How long do you usually use Oxygen treatment for headache attacks?

- How many liters per minute of Oxygen do you take during Oxygen treatment?

- Do you know how many liters of Oxygen per minute officially advice to be used during Oxygen treatment?
